# Insights into body size variation in cetaceans from the evolution of body-size-related genes

**DOI:** 10.1186/s12862-019-1461-9

**Published:** 2019-07-27

**Authors:** Yingying Sun, Yanzhi Liu, Xiaohui Sun, Yurui Lin, Daiqing Yin, Shixia Xu, Guang Yang

**Affiliations:** Jiangsu Key Laboratory for Biodiversity and Biotechnology, College of Life Sciences, Nanjing Normal University, Nanjing, 210023 China

**Keywords:** Cetacean, Body size, Body-size-related genes, Positive selection

## Abstract

**Background:**

Cetaceans exhibit an exceptionally wide range of body size, yet in this regard, their genetic basis remains poorly explored. In this study, 20 body-size-related genes for which duplication, mutation, or deficiency can cause body size change in mammals were chosen to preliminarily investigate the evolutionary mechanisms underlying the dramatic body size variation in cetaceans.

**Results:**

We successfully sequenced 20 body-size-related genes in six representative species of cetaceans. A total of 46 codons from 10 genes were detected and determined to be under strong positive selection, 32 (69.6%) of which were further found to be under radical physiochemical changes; moreover, some of these sites were localized in or near important functional regions. Interestingly, positively selected genes were well matched with body size evolution: for small cetaceans, strong evidence of positive selection was detected at *ACAN, OBSL1,* and *GRB10*, within which mutations or duplications could cause short stature; positive selection was found in large cetaceans at *CBS* and *EIF2AK3*, which could promote growth, and at the *PLOD1* gene, within which mutations could cause tall stature. Importantly, relationship analyses revealed that the evolutionary rate of *CBS* was positively related to body length and body mass with statistical significance. Additionally, we identified 32 cetacean-specific amino acid changes in 10 genes.

**Conclusions:**

This is the first study to investigate the molecular basis of dramatic body size variation in cetaceans. Our results provide evidence of the positive selection of several body-size-related genes in cetaceans, as well as divergent selection between large or small cetaceans, which suggest cetacean body size variation possibly associated with these genes. In addition, cetacean-specific amino acid changes might have played key roles in body size evolution after the divergence of cetaceans from their terrestrial relatives. Overall, the evolutionary pattern of these body-size-related genes could provide new insights into genetic mechanisms for the body size variation in cetaceans.

**Electronic supplementary material:**

The online version of this article (10.1186/s12862-019-1461-9) contains supplementary material, which is available to authorized users.

## Background

Fossils have revealed that cetaceans (whales, dolphins, and porpoises) originated 56–53 Ma from terrestrial artiodactyl ancestors [1]. Extant cetaceans can be subdivided into two suborders (Odontoceti and Mysticeti) within a wide range of marine habitats from coastal to oceanic and from tropical to polar [2]. As a result of lifestyle changes, cetaceans presented many extreme physiological and morphological adaptations among mammals, such as the loss of hindlegs, forelimbs that changed into flippers, lack of hair coats, thick blubber, feeding transition from herbivorous to carnivorous, and the loss of nearly all taste receptors, as well as the development of underwater sensory systems [3–6]. Benefiting from advances in cetacean whole genome sequencing projects, the molecular mechanisms underlying those major evolutionary changes, e.g., thickening of blubber, loss of hair, feeding habit transitions, brain size enlargements, and hypoxia tolerance [7–12], are beginning to be understood.

One of the most remarkable external changes in cetacean evolution is the dramatically wide range of body size, from less than 2 m in length and less than 50 kg in weight for porpoises to over 30 m and more than 150 tons for blue whales (*Balaenoptera musculus*), with more than a 20-fold increase in body length and a 3500-fold increase in body mass. Cetacean body size seems to have increased over evolutionary time, which follows Cope’s rule [13]. Moreover, the evolutionary rate of body mass in cetaceans exceeds that of terrestrial mammals perhaps due to “aquatic weightlessness”, migratory behaviour, and selection related to thermoregulation and feeding ecology in an aquatic environment [14]. Among these factors, temperature was reported to affect the body size of cetaceans, in agreement with Bergmann’s rule that animals living in colder climates are generally larger than those living in warmer regions [15]. For example, the largest living animal, the blue whale, frequents Arctic and Antarctic waters in the respective summer seasons and moves to the warmer subtropics and tropics during winters. By contrast, dolphins and porpoises, generally much smaller than whales, e.g., vaquita *Phocoena sinus*, the smallest cetacean, is only 1.4 m in body length and 42 kg in body mass and usually lives close to shore in shallow water. Large body size has been demonstrated to have many advantages, such as enhancing predation success, suitability for a more generalist diet, and increasing tolerance to extreme environments, but it also has some weaknesses such as being more susceptible to extinction and having lower fecundity [16]. However, the genetic bases of dramatic body size variation in cetaceans remain poorly explored.

Body size is a typical quantitative or complex trait that shows continuous variation [17]. Previous studies have reported that many discrete genes are involved in individual development, genetic diseases, or body size regulation. It has been reported that some genes involved in promoting growth or mutations in genes could cause tall stature (e.g., gigantism) and overgrowth, such as aryl hydrocarbon receptor interacting protein (*AIP*), cystathionine β - synthase (*CBS*), Natriuretic peptide receptor 2 (*NPR2*), nuclear receptor binding SET domain protein 1 (NSD1), lysyl hydroxylase 1 (*PLOD1*, also *LH1*), pleomorphic adenomagene 1 (*PLAG1*), translation initiation factor2-α kinase 3 (*EIF2AK3*), G-protein-coupled receptor 101 (*GPR101*), N-acetylgalactosamine-6-sulfate-sulfatase (*GALNS*), multiple endocrine neoplasia type I (*MEN1*), and mediator complex subunit 12 (*MED12*), which have been called “tall stature-related genes”. In contrast, some genes involved in inhibiting growth or mutations in genes result in short stature (e.g., dwarfism), including aggrecan (*ACAN*), obscurin-like 1 (*OBSL1*), growth factor receptor-bound protein 10 (*GRB10*), pituitary specific transcription factor 1 (*PIT-1*), Kir inward rectifier potassium channels (*KCNJ2*), Noggin (*NOG*), cyclin-dependent kinase inhibitor 1B (*CDKN1B*), and glypican-3 (*GPC3*), which have been called “short stature-related genes”. In addition, some genes, such as Fibrillin-1 (*FBN1*), have been associated with both overgrowth and dwarfism, depending on the kinds of changes that occurred on them; mutations in this gene have been described in Marfan syndrome, which is characterized by tall stature and arachnodactyly, whereas the TB5 mutations in this gene were responsible for short stature phenotypes. These genes, due to their association with body size, are termed body-size-related genes in the present study, and detailed function information is listed in (see Additional file 1: Table S1).

In the present study, coding sequences of the above 20 candidate genes were examined in cetaceans of both large and small body size to explore the evolutionary patterns and their association with the morphological variables of body size. Our results provide evidence of the positive selection and cetacean-specific amino acid changes of body-size-related genes in cetaceans, as well as divergent selection between large or small cetaceans, which suggested body size variation in cetaceans possibly associated with these genes.

## Results

### Positive selection of body-size-related genes in cetaceans

We successfully sequenced 20 body-size-related genes in six representative species of cetaceans: Omura’s baleen whales *Balaenoptera omurai*, striped dolphins *Stenella coeruleoalba*, pantropical spotted dolphins *S. attenuata*, common dolphins *Delphinus delphis*, Risso’s dolphins *Grampus griseus*, and dwarf sperm whales *Kogia simus*. Newly sequenced genes (GenBank accession nos. MH729659-MH729778) covered at least 80% of the full CDS. In addition, the orthologous genes of the 20 body-size-related genes were downloaded from another 10 cetacean species from their published database (see Additional file 1: Table S2). Preliminary alignment of gene sequences showed no frame shift mutations or premature stop codons. The one-ratio model, which assumes that one ratio occurs across the phylogenetic tree, showed that the ω ratios of 20 body-size-related genes ranged from 0.009 to 0.332 (see Additional file 1: Table S3), which indicates that strong purifying selection acts on these genes to constrain their important functions in body size development. A pair of site models (M8 VS M8a), implemented in the CODEML program of PAML 4.7 [18], was further used to test positively selected sites in the cetacean-only dataset. The likelihood ratio test (LRT) showed that M8 fitted the data better than M8a at 10 genes (i.e., *ACAN*, *AIP*, *CDKN1B*, *EIF2AK3*, *FBN1*, *MED12*, *MEN1*, *NPR2*, *NSD1*, and *OBSL1*), with 65 codons identified to be under positive selection using the BEB approach with posterior probabilities ≥0.85 (Table 1). Significant evidence of positive selection was further corroborated by the other two ML methods (FEL and REL) implemented in Datamonkey. Sixteen codons from 7 genes (*ACAN*, *AIP*, *CDKN1B*, *EIF2AK3*, *FBN1*, *MED12*, and *NPR2*) and 61 codons from 8 genes (*ACAN*, *AIP*, *EIF2AK3*, *FBN1*, *MED12*, *MEN1*, *NSD1*, and *OBSL1*) were examined to be under positive selection by REL and FEL, respectively (Table 1). A total of 46 positively selected sites were thus identified in 10 genes (i.e., *ACAN, AIP, CDKN1B, EIF2AK3, FBN1, MED12, MEN1, NPR2, NSD1, OBSL1*) by at least two ML methods and were thus regarded as robust candidates for positively selected sites. Thirty-two (69.2%) of them were found to have radical amino acid changes detected using a complementary protein-level approach implemented in TreeSAAP (see Additional file 1: Table S4), which provided additional evidence for positive selection in cetaceans.

**Table 1:** Genes and sites inferred to be under positive selection using three ML methods

Genes	PAML					Datamonkey		
	-Ln(M8a)	-Ln(M8)	-2△Lnl (*p*-value)	ω value	Positively selected sites (*P* ≥ 85%)^a^	FEL (*p* < 0.1)^b^	REL (*p* > 50)^c^	% of Sites ^d^
*ACAN*	(16 sequences)							
(2022aa)	13,517.793	13,357.328	320.930 (<0.001)	11.434	***9***, ***360***, ***703***, 806, **975**, ***980***, ***985***, 992, 1002, 1068, **1100**, 1185, 1270, 1411, 1532, 1626, 1710, **1734**, **1944**	***9***, **146**, ***360***, **407**, ***703, 980, 985***	***9***, **146**, 219, ***360***, 396, **407**, 686, ***703***, **975**, ***980***, ***985***, 1016, **1100**, 1567, **1734**, 1750, **1944**	0.544(11)
*AIP*	(14 sequences)							
(330aa)	1859.035	1855.058	7.953 (<0.005)	4.488	***43***, **45**, **78**, 86, ***131***, 218	***43***, ***131***	***43***, **45**, 56, **78**, ***131*,** 301	1.212(4)
*CDKN1B*	(16 sequences)							
(198aa)	1015.965	1013.544	4.841	21.427	**162**	**162**		0.505(1)
*EIF2AK3*	(16 sequences)							
(964aa)	5985.966	5976.756	18.42 (<0.001)	3.978	***154***, 358, 558, 628, **677**, **897**	***154***	***154***, **677**, 795, **897**	0.311(3)
*FBN1*	(15 sequences)							
(2871aa)	15,888.036	15,872.839	30.393 (<0.001)	3.387	300, **2443**, **2696**, **2699**, ***2701***, **2737**, ***2741***	**1946**, ***2701***, ***2741***	120, 1175, **1946**, 2336, **2443**, **2696**, **2699**, ***2701***, **2737**, ***2741***, 2775	0.244(7)
*MED12*	(16 sequences)							
(2050aa)	9844.493	9840.242	8.502 (<0.005)	12.298	312, ***1621***	***1621***	***1621***	0.049(1)
*MEN1*	(16 sequences)							
(610aa)	3657.161	3628.394	57.534 (<0.001)	7.403	**526**, 536, **542**, 594		394, 459, 475, **526**, **542**	0.328(2)
*NPR2*	(16 sequences)							
(1047aa)	5716.770	5687.387	58.825 (<0.001)	14.294	**325**	**325**		0.096(1)
*NSD1*	(16 sequences)							
(2696aa)	13,297.254	13,276.127	42.253 (<0.001)	4.399	**111**, **652**, **964**, **978**, 1144, **1493**, **1518**, **1853**, **2236**, **2264**, **2335**, **2411**		**111**, **652**, **964**, **978**, **1493**, **1518**, **1853**, **2236**, **2264**, **2335**, **2411**	0.408(11)
*OBSL1*	(16 sequences)							
(1729aa)	10,250.581	10,242.647	15.868 (<0.001)	4.348	**638**, **1242**, **1395**, 1423, **1428**, 1564, **1617**		592, **638**, **1242**, **1395**, **1428**, **1617**	0.289(5)

Note: The codons identified to be under positive selection by at least two or three ML methods are marked in bold and "bold and Italic", respectively.

^a^Codons identified by M8 model in PAML using a Bayes Empirical Bayes (BEB) analysis with posterior probabilities ≥85%

^b^Codons detected by FEL implemented in Datamonkey web server with significance levels of 0.1

^c^Codons determined by REL in Datamonkey web server with Bayes factors >50

^d^No. of sites indicate positively selected sites identified by both ML method

**Fig. 1:**
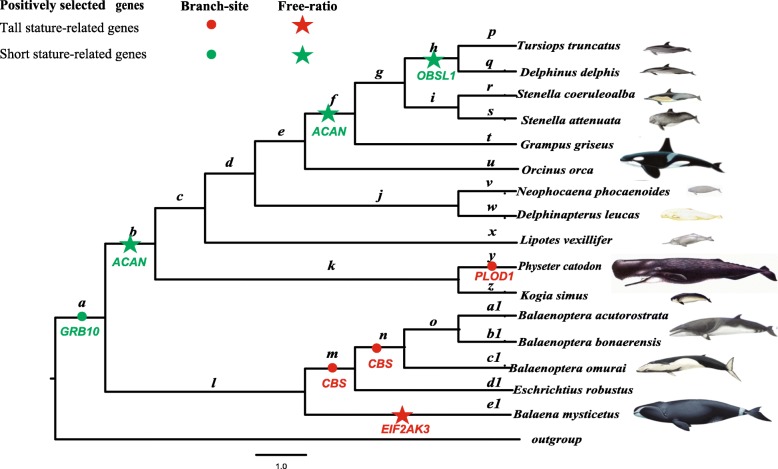
Evidence of positive selection across the phylogeny of cetaceans identified by the free-ratio and branch-site models. Positive selection across the cetacean phylogeny is marked with different colours: tall stature-related genes (red), short stature-related genes (green)

To test whether positive selection was restricted to some specific lineages, the free-ratio and branch-site models were used in our analysis. LRT revealed that the free-ratio model fitted the data better than the one-ratio model at 11 genes (i.e., *ACAN*, *AIP*, *EIF2AK3*, *FBN1*, *GALNS*, *GRB10*, *MED12*, *MEN1*, *OBSL1*, *PLAG1*, and *PLOD1*) (Additional file 1: Table S3). Specifically, evidence of positive selection was determined along the lineage to the last common ancestor (LCA) of Odontoceti and LCA of *G. griseus* at *ACAN*, the LCA of *T. truncatus* and *D. delphis* at *OBSL1*, as well as the terminal branch of *B. mysticetus* at *EIF2AK3* (Fig. 1). Moreover, the more stringent branch-site model revealed that positive selection was identified along the LCA of *Balaenopteridae* and LCA of *E. robustus* at *CBS*, the LCA of cetaceans at *GRB10*, as well as the lineage leading to *Physeter catodon* at *PLOD1* after FDR correction for multiple tests. In addition, four codons were identified to be under positive selection by the BEB procedure (Fig. 1).

**Fig. 2:**
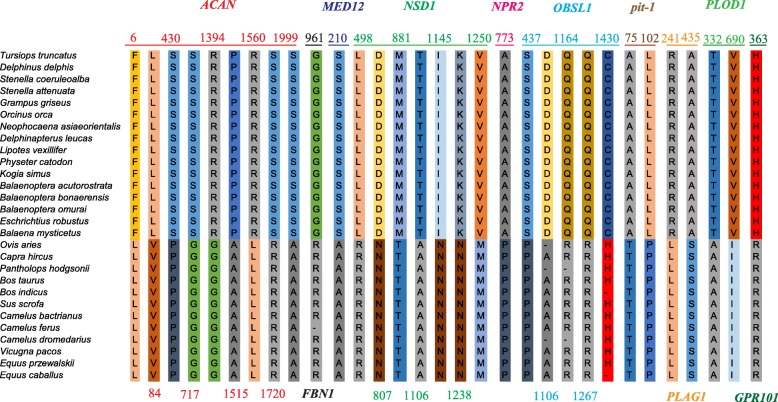
Special sites of cetaceans when compared with related terrestrial mammals

We classified representative cetaceans into small cetaceans and large cetaceans to explore whether cetaceans with divergent body size evolved under different evolutionary pressure. The ω values estimated for small cetaceans were almost twice as high as those in the large cetaceans at four genes, i.e., *FBN1*, *GRB10*, *NPR2* and *NSD1* (see Additional file 1: Table S5). Additionally, compared with terrestrial mammals, 32 cetacean-specific amino acid changes were identified at 10 genes (*ACAN*, *FBN1*, *GPR101*, *MED12*, *NPR2*, *NSD1*, *OBSL1*, *PIT-1*, *PLAG1*, *PLOD1*) (Fig. 2).

### Spatial distribution of positively selected sites and cetacean-specific sites in protein structures

To gain insight into the functional significance of the putative positively selected sites and cetacean-specific sites, a total of 46 radical amino acid changes subjected to positive selection and 32 cetacean-specific sites were mapped onto the 3D structures of the corresponding proteins. It was observed that many positively selected sites and cetacean-specific sites were located in or close to the functional regions (see Additional file 2: Figure S1). For example, one positively selected site of *AIP* (site 78) was located in N-terminus immunophilin-like domains, and missense mutation of several sites in this domain were reported to cause gigantism (e.g., V49 M, R56C, K58 N, E84K). In addition, radical change of amino acids was detected at residue 162 by two ML methods in the *CDKN1B* gene. 3D analysis showed that the site was located in the nuclear localization signal, which may contribute to its recognition with cytosolic nuclear transport receptors and that radical change in site 162 may help the protein in cell nuclei be more efficient and have the function of negative regulation of cell growth. Several radical changes subject to positive selection of *ACAN*, such as 975, 980, 985, were located in the chondroitin sulfate-rich domain (CS1), which is composed of repeats of nineteen amino acids and provides the aggrecan with its high anionic charge. For cetacean-specific sites, site 435 of *PLAG1* was located in the C-terminal serine-rich transactivation domain, which possesses a phosphorylation site and raises the possibility that phosphorylation may regulate the transactivation capacity of *PLAG1*. Another gene, *OBSL1*, also showed some cetacean-specific sites, among them, site 437 located in fibronectin-like (Fn3) domains, sites 1106 and 1164 located in immunoglobulin (Ig-like 11) and site 1267 located in the Ig-like 12 domain.

### Association between gene evolution and morphological variables

**Fig. 3:**
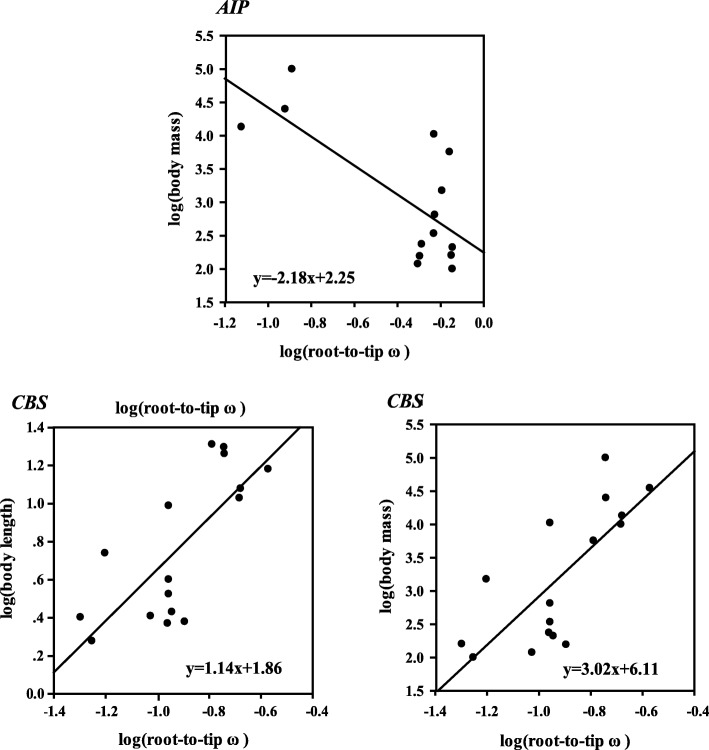
Regression analyses between root-to-tip ω and morphological variables (body length and body mass)

It has been regarded that tests for statistical association between genes and normal variation in phenotypes is a good strategy for examining the phenotypic consequences of a signature of ongoing selection [19]. We performed phylogenetic generalized least squares (PGLS) regressions to explicitly address the link between evolutionary rate of each gene under positive selection and body length/body mass. Regression analyses revealed significant positive association between log (root-to-tip ω) and log (body length) at the *CBS* (*R*^2^ = 0.551, *P* = 0.014) and between log (root-to-tip ω) and log (body mass) at the *CBS* (*R*^2^ = 0.561, *P* = 0.017). However, a significant negative association between log (root-to-tip ω) and log (body mass) was tested at the *AIP* gene (R^2^ = 0.522, *P* = 0.004, Fig. 3), whereas no such association was detected for other genes under positive selection (see Additional file 1: Table S6).

## Discussion

As is well known, modern cetaceans show an exceptionally wide range of body size, including the largest animal ever to live on the earth, the blue whale, and some small dolphins and porpoises, with a 20-fold body size difference and a 3500-fold mass difference. Previous studies have revealed clear evidence that the ancestor of odontocetes and mysticetes had a body size similar to that of the adult living bottlenose dolphin [20]. During the adaptive radiation, body size increased in mysticetes, whereas odontocetes exhibited a general decrease in body size (except for the sperm whale with increased body size), which was regarded to be related to their special dietary strategy, diving depth, temperature, and other factors [20, 21]. It is widely accepted that body size variation during cetacean evolution is the product of rapid divergence into new adaptive zones. Our study showed clear evidence that different evolutionary patterns of body-size-related genes, investigated in cetacean clades with contrasting body size, might contribute to an understanding of body size evolution at the molecular level.

### Positive selection in cetacean clades with contrasting body size

A total of 46 codons from 10 body-size-related genes were detected to be under positive selection by at least two ML methods, 32 (69.2%) of which were further found to be under radical physiochemical changes; some of these sites were localized in or near the important functional regions of proteins. Interestingly, positive selection of body-size-related genes identified in cetacean clades were well matched with their contrasting body size. Positive selection detected at *ACAN*, *OBSL1* and *GRB10*, whose mutations or duplications could cause short stature in mice, could provide insights into body decrease along lineages of small cetaceans, whereas positive selection at *CBS*, *PLOD1* and *EIF2AK3* in large cetacean genes could be explained by their role in promoting growth and causing body increase and tall stature.

Strong evidence of positive selection was found to act on the *ACAN* gene in odontocetes, which has a relatively smaller body size. Three codons (975, 980, 985) especially under positive selection were located in the chondroitin sulfate-rich domain (CS1), which is composed of repeats of nineteen amino acids and provides the aggrecan with its high anionic charge. In addition, some sites were found to be under radical change in the amino acid property. For example, position 985 of *ACAN* was identified to have a change in the “alpha-helical tendencies” property that would lead to a more rigid alpha helical form, which would help provide a more stable lipid raft composition [22]. Therefore, we speculated that the amino change of this site might play a crucial effect for the structure and function of protein. Significant positive selection in *ACAN* was subsequently examined in the common ancestor branch of Odontoceti, which was characterized by decreases in body mass [14]. *ACAN* encodes aggrecan, a proteoglycan in the extracellular matrix of the growth plate and other cartilaginous tissues, which is essential for cartilage structure. *ACAN* gene mutation could cause bulldog dwarfism in Dexter cattle due to the absence of normal aggrecan [23]. For humans, a single base pair insertion in *ACAN* resulted in spondyloepiphyseal dysplasia type Kimberly, characterized by shortened limbs and trunk [24]. Taken together, positive selection identified in cetaceans at the *ACAN* gene suggested this gene were possibly associated with restricting the body size increase during the evolution of small cetaceans.

Similar results were also found in the *GRB10*, a growth suppressor. It was found that the evolutionary rate of *GRB10* for small cetaceans was almost twice as high as that of large whales. More importantly, branch-site analysis revealed significant positive selection in the ancestor of cetaceans that was estimated to evolutionarily decrease body mass [14]. Previous experiments confirmed that overexpression of *GRB10* in human or transgenic mice could cause postnatal growth restriction [25] and that loss of its function in mice resulted in foetal and placental overgrowth [26]. The higher evolutionary rate of *GRB10* in small cetaceans and positive selection in the lineages with decreased body mass would thus help to restrict body size growth. *OBSL1* is a cytoskeletal adaptor protein, and mutation of this gene was confirmed to cause the primordial growth disorder 3-M syndrome in humans [27]. Similarly, positive selection acting on the *OBSL1* was identified in the last common ancestor of both small dolphins, i.e., the bottle-nosed dolphin and the common dolphin, which implies that the evidence of positive selection in this gene might play roles in restricting the body size increase of small cetaceans.

In contrast, significant positive selection was identified in the large whales at the *CBS*, *PLOD1*, and *EIF2AK3* genes that might promote body increase. Positive selection acting on the *CBS* gene was detected in both branch *m*, with large body length up to 15 m and body mass up to 35,000 kg, and branch *n*, with large body length up to 12 m and body mass up to 135,000 kg (Fig. 1). Moreover, our association analyses showed that the *CBS* evolutionary rate was significantly positively related to body length and body mass. This result is consistent with the fact that *CBS*-deficient (*Cbs*^*−/−*^) mice displayed severe growth retardation [28]. Interestingly, evidence of positive selection was also determined in the lineage leading to the largest toothed whale, i.e., the sperm whale (20.5 m in length and 57,000 kg in mass) in the *PLOD1* gene, which has been previously shown to cause the Nevo syndrome, clinically characterized by increased growth, kyphosis, a prominent forehead, and other factors [29]. Previous studies showed that loss-of-function mutations in *EIF2AK3* resulted in Wolcott-Rallison Syndrome in humans and that *EIF2AK3*-deficient (*Perk*^*−/−*^) mice also exhibited skeletal dysplasias at birth and postnatal growth retardation [30, 31]. Notably, significant positive selection in the *EIF2AK3* gene was examined in the lineages leading to the bowhead whale (19.8 m in length and 100,000 kg in mass), which suggests that this gene may contribute to the tremendous body size of the bowhead whale. Notably, the function of these body-size-related genes used in our study are forecasted according to mutations from human diseases and experiment of knock-out mice. Considering these genes are pleiotropic, we should do functional experiment in future to test whether these positive selected sites paly a key role in cetacean body size variation.

### Cetacean-specific amino acid change provides evidence of adaptive evolution

Compared with terrestrial mammals, 32 cetacean-specific amino acid changes in 10 genes were identified; 26 of them (81.2%) were identified to be under radical changes, and some of them were also located in or close to important functional regions. For example, one cetacean-specific amino acid site (773) identified to be under radical change at the *NPR2* gene was located in the kinase homology domain of the *NPR2*. It was reported that the *NPR2* gene positively regulates longitudinal bone growth. Loss-of-function mutations of *NPR2* cause short stature, and, conversely, gain-of-function mutations are associated with an overgrowth disorder [32]. For *ACAN*, nine cetacean-specific amino acid changes were found and six of them were identified to be under radical changes. Among them, site 717 was located in the KS domain, which could enhance the load-bearing capability of aggrecan in cartilage and may contribute to tissue development. Sites 1394, 1426, 1515, 1560 and 1720 were located in the CS2 domain, and negatively charged chondroitin sulfate chains in this domain account for the major function of aggrecan as a structural proteoglycan. Note that seven positively selected sites (i.e., 1100, 1411, 1532, 1626, 1710, 1734, 1944) were also located in this CS2 domain. In another example, one cetacean-specific acid site (75) was identified in the *PIT-1* gene, which is a transcription factor responsible for anterior pituitary development and pituitary-specific gene expression [33]. Mutations in this gene were first observed in Snell and Jackson dwarf mice because of pituitary hormone deficiency (CPHD). Previous studies have shown that mutations in codon 75 (T → A) could increase its interaction with the *LHX3* gene, which encodes LIM homeodomain class transcription factors that have important roles in pituitary and nervous system development. Interestingly, an A (alanine) is present in all cetaceans, whereas a T (threonine) is present in other terrestrial mammals. Notably, change in site 76 (P → L) results in CPHD1, which reduces transactivation capacity on the *GH1* gene, increases the functional binding on the *GH1* promoter, and increases the interaction with *ELK1*, *LHX3* and *PITX1*. Therefore, our results imply that these special sites might play key roles in body size evolution after the divergence of cetaceans from their terrestrial relatives. Of course, further investigation of the site-directed mutagenesis of these important sites is necessary in the future to confirm their role in body size evolution.

## Conclusions

Cetaceans show an exceptionally wide range of body size, which has been regarded to be related to their special dietary strategy, diving depth, temperature, and other factors. In this paper we present the first systematic investigation of 20 body size-related genes of representative cetacean lineages. Our results provide evidence of the positive selection of several body-size-related genes in cetaceans, as well as divergent selection between large and small cetaceans, which suggest their contribution to body size variation in cetaceans. Moreover, cetacean-specific amino acid changes might have played key roles in body size evolution after the divergence of cetaceans from their terrestrial relatives. Overall, the evolutionary pattern of these body-size-related genes could provide new insights into genetic mechanisms for body size variation in cetaceans. Importantly, some crucial codons detected in this study, including positively selected sites and cetacean-specific sites, provide a basis for function tests in the future.

## Methods

### Body-size-related genes acquisition

The coding sequences of 20 body-size-related genes were first screened and downloaded from the genomes of ten cetacean species: bottlenose dolphins *T. truncatus*, killer whales *Orcinus orca*, baiji *Lipotes vexillifer*, Yangtze finless porpoises *Neophocaena asiaeorientalis*, beluga whales *Delphinapterus leucas*, sperm whales *Physeterm acrocephalus*, bowhead whales *Balaena mysticetus*, minke whales *B. acutorostata*, Antarctic minke whales *B. bonaerensis*, and grey whales *Eschrichtius robustus* (see Additional file 1: Table S2). Low-quality and/or low-integrity gene sequences were further confirmed by searching from genome sequences of relevant cetaceans using bottlenose dolphin genes as queries to the BLAST (basic local alignment search tool) algorithm. Then, we amplified and sequenced these 20 genes in another six cetacean species: *B. omurai*, *S. coeruleoalba*, *S. attenuata*, common dolphins *D. delphis*, *G. griseus*, and *K simus*. The six cetacean samples were collected from dead individuals in the wild; no ethics statement is required for such occasions. Genomic DNA extraction, polymerase chain reaction (PCR) amplification, and sequencing were conducted as described in Xu et al. [11]. The PCR primers are listed in (see Additional file 3: Table S7).

The orthologous genes of each candidate gene were also downloaded from twelve terrestrial relatives: cows *Bos taurus*, zebu cattle *Bos indicus*, sheep *Ovis aries*, goats *Capra hircus*, Tibetan antelopes *Pantholops hodgsonii*, camels *Camelus ferus*, bactrian camels *Camelus bactrianus*, Arabian camels *Camelus dromedarius*, alpacas *Vicugna pacos*, pigs *Sus scrofa*, Przewalski’s horses *Equus przewalskii*, and horses *Equus caballus* (Additional file 1: Table S2). For genomic DNA, intron-exon boundaries were recognized from strict conserved splice signals (GT/AG), and the exons of each gene were concatenated according to the coding sequences of the known relative species. The nucleotide sequences and their deduced amino acid sequences of each gene were aligned using Muscle in MEGA6 [34] and verified by visual inspection (see Additional file 4).

### Molecular evolutionary analysis

Comparisons of nonsynonymous (*d*_N_)/synonymous (*d*_S_) substitution ratios have become a useful means for quantifying the impact of natural selection on molecular evolution [35, 36]. Values of ω < 1, = 1, and > 1 correspond to purifying selection, neutral evolution, and positive selection, respectively. The codon-based maximum likelihood models implemented in the CODEML program of PAML 4.7 [18] were applied to estimate the ω values. To identify the probabilities of sites under positive selection in each gene, two pairs of site models: M8a (beta & ω2 = 1) versus M8 (beta & ω2 > 1), for which ω could vary among sites were implemented in the cetacean-only dataset. LRT statistic (2△L), which approximates a chi-square distribution, was used to compare nested likelihood models. Positively selected sites were identified using BEB analysis with posterior probabilities of ≥0. 85. Considering that ω values estimated by PAML models only allow for variation in the nonsynonymous substitution rate, and positively selected sites were further evaluated by fixed-effect likelihood (FEL) and random effect likelihood (REL) models implemented in the Datamonkey web server, which incorporated variation in the rate of synonymous substitution [37]. The REL approach allows variation in nonsynonymous and synonymous rates across sites according to a predefined distribution, and the FEL method directly estimates nonsynonymous and synonymous substitution rates at each site [38]. Therefore, sites with a significance level < 0.1 for FEL or a Bayes factor > 50 for REL were regarded as candidates for selection.

To test whether positively selected sites were limited to a specific lineage, the branch-site model and free-ratio model implemented in the CODEML program of PAML 4.7 were used in the all-mammals dataset. The branch-site model can detect positive selection at specific sites along a specific branch [39], whereas the free-ratio model allows an independent ω value for each branch [18]. A false discovery rate (FDR) correction for multiple tests was conducted in the branch-site model analysis [40]. BEB analysis was also used to test positively selected sites with posterior probabilities of ≥0.85 in the branch-site model. Three starting ω values (0.4, 1, and 2) were used to check for the existence of multiple local optima.

To determine if different selection pressures were acting on cetacean clades with contrasting body size, we divided sixteen cetacean species into large-bodied cetaceans (body length > 6 m), including bowhead whales, minke whales, Antarctic minke whales, Omura’s baleen whales, grey whales, sperm whales and killer whales, and small cetaceans (such as bottlenose dolphins, baiji and finless porpoises) according to the recommendation of Weber [41]. Branch models that allow different branches to have different ω, the so-called ‘two ratio’ and ‘three ratio’ models, were implemented in CodeML and were used to evaluate the ω values between the large and small cetacean groups. First, we set up foreground (particular lineages of interest) and background lineages (the remaining lineages). The one-ratio model, which enforces the same ω ratio for all lineages, was compared with the two-ratio model that allows one ω ratio for all branches of cetaceans and another for all terrestrial mammal branches. Moreover, we used the three-ratio model, which assumes that large cetaceans, small cetaceans and terrestrial mammals have independent ω values, to investigate whether different selective pressures were imposed on different types of cetaceans. All nested models were compared using LRTs.

To detect significant physicochemical amino acid changes in each gene, we used the algorithm implemented in the TreeSAAP 3.2 software package [42], which measures the selection based on 31 structural and biochemical amino acid property changes. According to the change in specific physicochemical properties, the magnitudes of non-synonymous changes are classified into eight categories from conservative (1–3) to very radical substitutions (6–8). A z-score was calculated for each category. Only significant positive z-scores in radical substitutions were considered to be affected by positive destabilizing selection.

### Mapping of positively selected sites and cetacean-specific sites onto protein structures

To gain insights into the functional significance of the putative positively selected sites and cetacean-specific sites, we mapped these sites onto the crystal structures. The three-dimensional (3D) structures of each gene subject to positive selection and cetacean-specific sites were constructed by using I-TASSER server [43], a hierarchical protein structure modelling approach based on the secondary structure enhanced profile–profile threading alignment and the iterative implementation of the TASSER program. Additionally, the functional information of genes under positive selection was derived from UniProt (http://www.uniprot.org/).

### Association analysis between gene evolution and phenotypes

To explore the potential relationships between the evolutionary rate (ω) of genes and body size phenotypes, we used the root-to-tip ω, which includes more evolutionary history of a locus as the evolutionary rate according to the method suggested by Montgomery et al. [44]. The branch models were used to estimate the average ω from the ancestral cetacean to each terminal species implemented in the CODEML program in PAML 4.7 [18]. The phenotypic traits, including body length and body mass of representative species of cetaceans, were collected from previously published data (see Additional file 5: Table S8). Phylogenetic generalized least squares (PGLS) regression was used to analyse the relationship between log-transformed (root-to-tip ω) and each log-transformed morphological variable. The lambda (λ) value estimated by the maximum likelihood method was used as a quantitative measure of phylogenetic signals [45]. All statistical analyses were performed using R 3.4.3 in the package Caper [46, 47].

## Additional files

Additional file 1: **Table S1.** Twenty body-size-related genes and their functions. **Table S2.** Sequence data used in this study, including taxonomy and accession numbers or Emsemble ID. **Table S3.** One-ratio model, Free-ratio model and Branch-site model analysis in 20 body-size-related genes. **Table S4.** Radical amino acid sites under positive selection detected by PAML, Datamonkey and TreeSAAP. **Table S5.** Log likelihood and omega values estimates under different branch models according contracting body length of cetaceans. **Table S6.** Association analysis between gene evolution and phenotypes. (DOCX 107 kb)

Additional file 2: **Figure S1.** Radical amino acid changes in selected sites and cetacean-special sites mapped on the three-dimensional structure of body-size-related genes. Sites marked with red balls stand for robust sites under selection and yellow balls stand for cetacean-special sites. The figures were created using PyMOL (http://www.pymol.org). (PDF 2961 kb)

Additional file 3: **Table S7.** Primer sets used to amplify the coding regions of body-size-related genes in this study (XLSX 25 kb)

Additional file 4: Alignment sequences of 20 body-size related genes used for this study. These files are Fast format, please use MEGA or Clustal to open them. (ZIP 133 kb)

Additional file 5: **Table S8.** Previously reported body size information for cetaceans. (DOCX 17 kb)

## Data Availability

All the data supporting our findings are contained within the manuscript and in the supplemental files.

## Abbreviations

3D: three-dimensional
*ACAN*: aggrecan
*AIP*: aryl hydrocarbon receptor interacting protein
BEB: Bayes Empirical Bayes
BLAST: basic local alignment search tool
*CBS*: cystathionine β – synthase
*CDKN1B*: cyclin-dependent kinase inhibitor 1B
*d*_N_: nonsynonymous substitution
*d*_S_: synonymous substitution
*EIF2AK3*: translation initiation factor2-α kinase 3
*FBN1*: Fibrillin-1
FDR: false discovery rate
FEL: fixed effects likelihood
*GALNS*: N-acetylgalactosamine-6-sulfate-sulfatase
*GPC3*: glypican-3
*GPR101*: G-protein-coupled receptor 101
*GRB10*: growth factor receptor-bound protein 10
*KCNJ2*: Kir inward rectifier potassium channels
LRT: likelihood ratio test
*MED12*: mediator complex subunit 12
*MEN1*: multiple endocrine neoplasia type I
*NOG*: Noggin
*NPR2*: Natriuretic peptide receptor 2
*NSD1*: nuclear receptor binding SET domain protein 1
*OBSL1*: obscurin-like 1
PCR: polymerase chain reaction
PGLS: phylogenetic generalized least squares
*PIT-1*: pituitary specific transcription factor 1
*PLAG1*: pleomorphic adenomagene 1
*PLOD1* (also LH1): lysyl hydroxylase 1 gene
REL: random effect likelihood
