# Supplementary material for: Insights into body size variation in cetaceans from the evolution of body-size-related genes
Source: BMC Evol Biol. 2019 Jul 27;19:157. doi: 10.1186/s12862-019-1461-9 (PMC6660953; doi:10.1186/s12862-019-1461-9)
Supplement: Supplementary file 1 — Table S1. Twenty body-size-related genes and their functions. Table S2. Sequence data used in this study, including taxonomy and accession numbers or Emsemble ID. Table S3. One-ratio model, Free-ratio model and Branch-site model analysis in 20 body-size-related genes. Table S4. Radical amino acid sites under positive selection detected by PAML, Datamonkey and TreeSAAP. Table S5. Log likelihood and omega values estimates under different branch models according contracting body length of cetaceans. Table S6. Association analysis between gene evolution and phenotypes. (DOCX 107 kb) [file 12862_2019_1461_MOESM1_ESM.docx]

**Electronic Supplementary Information (ESM)**

**Insights into body size variation in cetaceans from the evolution of body-size-related genes**

Yingying Sun^#^, Yanzhi Liu^#^, Xiaohui Sun, Yurui Lin, Daiqing Yin, Shixia Xu*, and Guang Yang*

Jiangsu Key Laboratory for Biodiversity and Biotechnology, College of Life Sciences, Nanjing Normal University, Nanjing 210023, China

* Corresponding authors; e-mail: xushixia78@163.com and gyang@njnu.edu.cn

Table S1 Twenty body-size-related genes and their function

| **Gene** | **Protein Product** | | **Function** | | **Diseases** | References | |
| --- | --- | --- | --- | --- | --- | --- | --- |
| **Tall stature-related genes** | | | | | | | |
| **CBS** | | Cystathionine β synthase | Catalyzes the condensation of homocysteine and serine to form cystathionine, a precursor of cysteine | clinical symptoms include ectopia lentis, vascular disease with life-threatening thromboembolisms, skeletal deformities, osteoporosis, and mental retardation. | | | Camp et al. 1983 |
| **NPR2** | | Natriuretic Peptide Receptor-B | Regulates skeletal growth | Loss-of-function mutations showed Short Stature, while gain-of-function mutations are associated with overgrowth | | | Bocciardi et al. 2007 |
| **PLAG1** | | pleomorphic adenoma  gene 1 | Involved in cell proliferation and differentiation | PLAG1 KO phenotype is reduced body size | | | Juma et al. 2016 |
| **EIF2AK3** | | translation initiation factor  2-α kinase 3 | Functions in maintaining the integrity of pancreatic β-cells, regulate protein translation | Wolcott-Rallison syndrome, growth retardation | | | Gupta et al. 2010 |
| **GPR101** | | G-protein-coupled receptor | Important for brain and pituitary  development | X-linked acro-gigantism syndrome, early-onset pituitary gigantism, short stature | | | Trivellin et al. 2014 |
| **GALNS** | | N-acetylgalactosamine-6-  sulfate-sulfatase | Derived from a cysteine residue by action of the formylglycine- generating enzyme | Morquio A disease, bone dysplasia, short trunk dwarfism | | | Tomatsu et al. 2010 |
| **MEN1** | | menin | Involved in transcriptional regulation, cell division and proliferation | Men1 knockout mice exhibit delayed development | | | Gao et al. 2008 |
| **AIP** | | Aryl hydrocarbon receptor  -interacting protein | Involved in various cellular pathways, function as a tumour suppressor gene | familial isolated pituitary  adenoma, gigantism,  acromegaly | | | Urbani et al. 2014 |
| **PLOD1** | | lysyl hydroxylase | Be responsible for hydroxylation of lysyl residues in collagen proteins | Nevo syndrome, tall stature | | | Visser et al. 2009 |
| **MED12** | | Mediator complex  Subunit 12 | Regulates signals involved in cell growth, development, and differentiation, RNA polymerase II transcription | X-linked intellectual disability syndromes, Opitz–Kaveggia syndrome, Lujan syndrome,  Ohdo syndrome | | | Graham & Schwartz 2013 |
| **NSD1** | | Histone-lysine  N-methyltranferase | Be essential for early post-implantation development | Sotos syndrome, multiple  myeloma and cancers | | | Opitz et al. 1998 |
| **Short stature-related genes** | | | | | | | |
| **CDKN1B** | cyclindependent kinase | | Relevant quences of modulating cell proliferation for tissue maintenance | | Cdkn1b-null mice grow to approximately twice the normal size and exhibit organomegaly. | Pruitt et al. 2013 | |
| **GRB10** | growth factor receptor –  bound protein 10 | | Mediates interactions between disparate proteins, role as a growth suppressor | | Loss of Grb10 function in the mouse results in fetal and placental overgrowth, overexpression of GRB10 results in SRS | Charalambous et al. 2003 | |
| **GPC3** | Glypican-3 | | Acts as an inhibitor of Hedgehog activity during development | | GPC3 null mice display developmental overgrowth. GPC3 TG mice showed smaller increase in liver weight than WT | Capurro et al. 2008 | |
| **NOG** | Noggin | | Involved in both bone and joint development | | Too little expression of NOG results in cartilage overgrowth and bony fusion. | Sara et al. 2008 | |
| **ACAN** | aggrecan | | Be essential for cartilage structure | | Bone development abnormalities in otherspecies, causing disproportionate dwarfism | Cavanagh et al. 2007 | |
| **OBSL1** | Obscurin-like 1 | | Functions as a cytoskeletal adaptor protein linking the nuclear proteins to the cytoplasmic support network | | 3-M syndrome, body growth restriction. | Demir et al. 2013 | |
| **PIT-1** | Pituitary-specific  transcription factor 1 | | Can activate the transcription of the growth hormone and prolactin promoters | | Snell dwarf phenotype | Cohen et al. 1995 | |
| **KCNJ2** | Kir2.1 | | Be an important contributor to the inward rectifier K^+^ current | | Andersen syndrome, skeletal developmental abnormalities | Plaster et al. 2001 | |
| **Genes related to both short stature and tall stature** | | | | | | | |
| **FBN1** | Fibrillin-1 | | Involved in the health of the heart and of the aorta interms of the cardiovascular system | | Marfan syndrome, Acromicric and Geleophysic Dysplasias. | Le Goff et al. 2011 | |

**Table S2** Sequence data used in this study, including taxonomy and accession numbers or Ensembl ID

| Genes/Species | **ACAN** | **AIP** | **CBS** | **CDKN1B** | **EIF2AK3** | | **FBN1** | **GALNS** |
| --- | --- | --- | --- | --- | --- | --- | --- | --- |
| Tursiops truncatus | This study | This study | This study | XM_004314783.2 | XM_019942241.1 | | XM_019929158.1 | XM_019923386.1 |
| Delphinus delphis | MH729741 | MH729772 | MH729695 | MH729774 | MH729673 | | MH729729 | MH729748 |
| Stenella coeruleoalba | MH729740 | MH729771 | MH729696 | MH729775 | MH729671 | | MH729725 | MH729743 |
| Stenella attenuata | MH729738 | MH729769 | MH729699 | MH729778 | MH729675 | | MH729728 | MH729746 |
| Grampus griseus | MH729739 | MH729767 | MH729700 | MH729776 | MH729672 | | MH729726 | MH729744 |
| Orcinus orca | XM_004278243.1 | XM_004278019.2 | XM_004264597.2 | XM_004281135.2 | XM_004285631.1 | | XM_004281306.1 | XM_012536827.1 |
| Neophocaena asiaeorientalis | XM_024766481.1 | XM_024749514.1 | XM_024758444.1 | XM_024733162.1 | This study | | XM_024740283.1 | XM_024735557.1 |
| Delphinapterus leucas | XM_022564016.1 | XM_022591258.1 | XM_022552874.1 | XM_022577630.1 | XM_022598936.1 | | XM_022587160.1 | XM_022582910.1 |
| Lipotes vexillifer | XM_007470511.1 | XM_007450425.1 | This study | XM_007469328.1 | XM_007466723.1 | | XM_007455563.1 | XM_007468440.1 |
| Physeter catodon | This study | XM_007126937.1 | XM_007127911.1 | XM_007120584.1 | XM_007102541.1 | | XM_007113096.1 | XM_007125507.1 |
| Kogia simus | MH729742 | MH729770 | MH729696 | MH729773 | MH729674 | | MH729730 | MH729747 |
| Balaenoptera bonaerensis | This study | This study | This study | This study | This study | | This study | This study |
| Balaenoptera acutorostrata | XM_007194745.1 | XM_007171072.1 | XM_007172678.1 | XM_007196051.1 | XM_007175315.1 | | XM_007170445.1 | XM_007172554.1 |
| Balaenoptera omurai | MH729737 | MH729768 | MH729698 | MH729777 | MH729676 | | MH729727 | MH729745 |
| Eschrichtius robustus | This study | This study | This study | This study | This study | | This study | This study |
| Balaena mysticetus | gnl\|BL_ORD_ID\|  14924 | gnl\|BL_ORD_ID\|  12844 | gnl\|BL_ORD_ID\|  448413 | gnl\|BL_ORD_ID\|  16442 | This study | | This study | gnl\|BL_ORD_ID\|  100930 |
| Ovis aries | XM_012098454.2 | XM_004019719.3 | XM_004003372.3 | XM_004006850.3 | XM_004005901.3 | | XM_012181624.2 | XM_012189671.2 |
| Capra hircus | XM_018066613.1 | XM_005699947.3 | XM_018051749.1 | XM_005680816.3 | XM_018055349.1 | | XM_018054172.1 | XM_018061859.1 |
| Pantholops hodgsonii | XM_005977186.1 | XM_005965721.1 | XM_005956789.1 |  | XM_005954891.1 | | XM_005958938.1 | XM_005956311.1 |
| Bos taurus | NM_173981.2 | NM_183082.2 | NM_001102000.2 | This study | XM_010810067.2 | | NM_174053.2 | NM_001206329.1 |
| Bos indicus | XM_019983198.1 | XM_019954449.1 | XM_019963159.1 | XM_019961532.1 | XM_019970273.1 | | XM_019967855.1 | XM_019979779.1 |
| Sus scrofa | NM_001164652.1 | XM_003122456.2 | XM_021071046.1 | AB031957.1 | XM_003124925.3 | | NM_001001771.1 | XM_005653234.2 |
| Camelus bactrianus | XM_010955056.1 | XM_010956731.1 | XM_010966562.1 | XM_010946445.1 | XM_010958963.1 | | XM_010965346.1 | XM_010968177.1 |
| Camelus ferus | XM_014565922.1 | XM_006175986.2 | This study | XM_006192124.2 | XM_006183595.2 | This study | | XM_014560417.1 |
| Camelus dromedarius | XM_010986843.1 | XM_010998125.1 | XM_010999672.1 | XM_010986237.1 | XM_010997866.1 | XM_010998625.1 | | XM_010998444.1 |
| Vicugna pacos | XM_015252199.1 | XM_006210704.2 | XM_006205036.2 | XM_006199874.2 | XM_006203818.2 | XM_015237098.1 | | XM_006212405.2 |
| Equus caballus | XM_014733894.1 | This study | XM_001490904.5 | This study | XM_014731045.1 | XM_001502259.5 | | XM_005608500.2 |
| Equus przewalskii | XM_008525424.1 | XM_008533869.1 |  | XM_008522926.1 | XM_008518670.1 | XM_008534321.1 | | XM_008508805.1 |
|  |  |  |  |  |  |  | |  |
| Genes/Species | **GPC3** | **GPR101** | **GRB10** | **KCNJ2** | **MED12** | **MEN1** | | **NOG** |
| Tursiops truncatus | XM_019919072.1 | XM_019919003.1 | This study | XM_004310954.2 | This study | This study | | XM_019945282.1 |
| Delphinus delphis | MH729723 | MH729754 | MH729732 | MH729690 | MH729669 | MH729706 | | MH729718 |
| Stenella coeruleoalba | MH729721 | MH729750 | MH729736 | MH729694 | MH729665 | MH729701 | | MH729713 |
| Stenella attenuata | MH729719 | MH729753 | MH729733 | MH729693 | MH729667 | MH729704 | | MH729716 |
| Grampus griseus | MH729720 | MH729751 | MH729735 | MH729691 | MH729666 | MH729702 | | MH729714 |
| Orcinus orca | XM_004285475.1 | XM_004278723.1 | XM_004277959.2 | XM_004275423.2 | XM_004275762.1 | XM_012537337.1 | | XM_004271794.2 |
| Neophocaena asiaeorientalis | XM_024746584.1 | XM_024766957.1 | XM_024764363.1 | XM_024760693.1 | XM_024758107.1 | XM_024749316.1 | | XM_024757637.1 |
| Delphinapterus leucas | XM_022554225.1 | XM_022554318.1 | XM_022593188.1 | XM_022589768.1 | XM_022561030.1 | XM_022591549.1 | | XM_022552602.1 |
| Lipotes vexillifer | XM_007462875.1 | XM_007452087.1 | XM_007448336.1 | XM_007454398.1 | XM_007470107.1 | XM_007462041.1 | | XM_007463875.1 |
| Physeter catodon | XM_007109879.1 | XM_007122545.1 | This study | XM_007120286.1 | XM_007114450.1 | This study | | XM_007109681.1 |
| Kogia simus | MH729724 | MH729749 | MH729731 | MH729689 | MH729668 | MH729705 | | MH729717 |
| Balaenoptera_bonaerensis | This study | This study | This study | This study | This study | This study | | This study |
| Balaenoptera acutorostrata | XM_007174525.1 | XM_007183682.1 | XM_007178346.1 | XM_007185592.1 | XM_007183630.1 | XM_007174289.1 | | XM_007176178.1 |
| Balaenoptera omurai | MH729722 | MH729752 | MH729734 | MH729692 | MH729670 | MH729703 | | MH729715 |
| Eschrichtius robustus | This study | This study | This study | This study | This study | This study | | This study |
| Ovis aries | XM_015105024.1 | XM_015105008.1 | XM_015095173.1 | XM_012109899.2 | XM_012106874.2 | XM_015103170.1 | | NM_001174110.1 |
| Pantholops hodgsonii | XM_005982382.1 | XM_005968598.1 | XM_005982788.1 | XM_005973168.1 | XM_005954830.1 | XM_005959320.1 | | XM_005985580.1 |
| Capra hircus | XM_018044068.1 | XM_005700428.2 | XM_018047456.1 | XM_005694470.3 | XM_018043874.1 | XM_018042892.1 | | XM_013971792.2 |
| Balaena mysticetus | gnl\|BL_ORD_ID\|  28049 | gnl\|BL_ORD_ID\|  15290 | gnl\|BL_ORD_ID\|42454 | gnl\|BL_ORD_ID\|  18997 | gnl\|BL_ORD_ID\|6538 | gnl\|BL_ORD_ID\|  67168 | | gnl\|BL_ORD_ID\|  17688 |
| Bos taurus | NM_001035463.2 | XM_015470205.1 | This study | NM_174373.2 | NM_001205878.1 | NM_001076161.2 | | XM_582573.9 |
| Bos indicus | XM_019956287.1 | XM_019955662.1 | XM_019959625.1 | XM_019981937.1 | XM_019955832.1 | XM_019954370.1 | | XM_019982609.1 |
| Sus scrofa | XM_013986449.1 |  | EF174198.1 | NM_214151.1 | XM_001927848.5 | XM_005660727.2 | | NM_001143691.1 |
| Camelus bactrianus | XM_010968452.1 | XM_010952526.1 | XM_010950213.1 | XM_010948607.1 | XM_010973669.1 | XM_010957098.1 | | XM_010951088.1 |
| Camelus ferus | XM_006194438.2 | XM_006194030.2 | XM_006178621.2 | XM_006194822.2 | This study | This study | | This study |
| Camelus dromedarius | XM_010989523.1 | XM_010993459.1 | XM_010999069.1 | XM_010975986.1 | XM_010978772.1 | XM_010994767.1 | | XM_010990519.1 |
| Vicugna pacos | XM_015249283.1 | XM_006218162.2 | XM_015249788.1 | XM_006199346.2 | XM_006217659.2 | XM_015249855.1 | | XM_006216420.1 |
| Equus caballus | XM_001488331.5 | XM_005614555.1 | XM_001498401.3 | KT381434.1 | XM_005614273.1 | XM_001916921.4 | | AF510666.1 |
| Equus przewalskii | XM_008526580.1 | XM_008511923.1 | XM_008538785.1 | XM_008522325.1 | XM_008511420.1 | XM_008532514.1 | | XM_008519820.1 |
|  | Table S2 (continued) | | |  |  |  | |  |
| Genes/Species | **NPR2** | **NSD1** | **OBSL1** | **PIT-1** | **PLAG1** | **PLOD1** | |  |
| Tursiops truncatus | This study | ENSTTRG00000009569.1 | This study | XM_004321205.1 | XM_019925318.1 | ENSTTRG00000003503.1 | |  |
| Delphinus delphis | MH729688 | MH729681 | MH729755 | MH729660 | MH729766 | MH729708 | |  |
| Stenella coeruleoalba | MH729683 | MH729682 | MH729759 | MH729664 | MH729761 | MH729707 | |  |
| Stenella attenuata | MH729686 | MH729678 | MH729757 | MH729663 | MH729764 | MH729710 | |  |
| Grampus griseus | MH729684 | MH729679 | MH729760 | MH729661 | MH729762 | MH729712 | |  |
| Orcinus orca | XM_004271418.2 | XM_004284816.2 | XM_004262669.1 | XM_004272645.1 | XM_004275001.1 | XM_004272408.1 | |  |
| Lipotes vexillifer | XM_007455974.1 | XM_007463086.1 | XM_007445798.1 | XM_007468998.1 | XM_007464041.1 | XM_007463175.1 | |  |
| Delphinapterus leucas | XM_022600157.1 | XM_022564373.1 | XM_022567778.1 | XM_022574705.1 | XM_022556823.1 | XM_022598493.1 | |  |
| Neophocaena asiaeorientalis | XM_024763024.1 | XM_024745593.1 | XM_024763139.1 | XM_024740848.1 | XM_024742644.1 | XM_024749759.1 | |  |
| Physeter catodon | XM_007108566.1 | XM_007102870.1 | XM_007122408.1 | XM_007102964.1 | XM_007117378.1 | XM_007129712.1 | |  |
| Kogia simus | MH729687 | MH729680 | MH729756 | MH729662 | MH729765 | MH729709 | |  |
| Balaenoptera_bonaerensis | This study | This study | This study | This study | This study | This study | |  |
| Balaenoptera acutorostrata | XM_007196983.1 | XM_007169770.1 | XM_007187997.1 | XM_007164092.1 | XM_007168591.1 | XM_007169960.1 | |  |
| Balaenoptera omurai | MH729685 | MH729677 | MH729758 | MH729659 | MH729763 | MH729711 | |  |
| Eschrichtius robustus | This study | This study | This study | This study | This study | This study | |  |
| Balaena mysticetus | gnl\|BL_ORD_ID\|  23636 | gnl\|BL_ORD_ID\|  472927 | gnl\|BL_ORD_ID\|21010 | gnl\|BL_ORD_ID\|  15661 | gnl\|BL_ORD_ID\|5766 | gnl\|BL_ORD_ID\|  20230 | |  |
| Ovis aries | XM_004004267.3 | XM_012178282.2 | This study | NM_001009350.1 | XM_004011687.3 | This study | |  |
| Capra hircus | NM_001285674.1 | XM_018050618.1 | XM_018058936.1 | NM_001285673.1 | XM_013969222.2 | XM_018060334.1 | |  |
| Pantholops hodgsonii | XM_005969999.1 | XM_005957947.1 | XM_005956700.1 | XM_005964353.1 | XM_005966435.1 | XM_005980810.1 | |  |
| Bos taurus | XM_010807963.2 | This study | NM_001075491.2 | NM_174579.4 | XM_005215433.2 | NM_174148.1 | |  |
| Bos indicus | XM_019966182.1 | XM_019964700.1 | XM_019977463.1 | XM_019967324.1 | XM_019974073.1 | XM_019976596.1 | |  |
| Sus scrofa | NM_001244322.1 | This study | XM_005672244.2 | HM163575.1 | XM_013996756.1 | XM_003127577.4 | |  |
| Camelus bactrianus | XM_010952855.1 | XM_010946200.1 | XM_010948839.1 | XM_010966454.1 | XM_010970685.1 | XM_010957787.1 | |  |
| Camelus ferus | XM_006187894.2 | This study | This study | XM_006187011.1 | XM_006191052.2 | This study | |  |
| Camelus dromedarius | XM_010995587.1 | XM_010992431.1 | XM_010988391.1 | XM_010978526.1 | XM_010989533.1 | XM_011000853.1 | |  |
| Vicugna pacos | XM_006204123.2 | XM_015234865.1 | XM_015241692.1 | XM_006215825.1 | XM_006204673.2 | XM_006196666.2 | |  |
| Equus caballus | XM_001504487.5 | XM_014730479.1 | XM_014856156.1 | XM_001501258.2 | XM_001497516.5 | XM_001491331.5 | |  |
| Equus przewalskii | XM_008508047.1 | XM_008542199.1 | XM_008507542.1 | XM_008507556.1 | XM_008522554.1 | XM_008522020.1 | |  |

Note: All the newly obtained sequences in our study were deposited in GenBank with accession numbers: MH729659-MH729778.

**Table S3** One ratio, Free-ratio model and Branch-site model analysis in 20 body-size-related genes

| **Genes** | **Models** | **-lnL** | **Models compared** | **2ΔInL** | **df** | **P value** | **ω value** | **Positively selected sites** |
| --- | --- | --- | --- | --- | --- | --- | --- | --- |
| **ACAN** | Branch model (Last common ancestral branch of *Odontoceti*, Last common ancestral branch of *Grampus griseus*) | | | | | | | |
|  | M0: One ratio | 24008.016 |  |  |  |  | ω **=**0.332 |  |
|  | M1: Free ratio | 23898.137 | M0 vs M1 | 110.068 | 53 | <0.001 | ω variation for each branch |  |
| **AIP** | Branch model | | | | | | | |
|  | M0: One ratio | 2893.084 |  |  |  |  | ω **=**0.062 |  |
|  | M1: Free ratio | 2812.754 | M0 vs M1 | 79.657 | 49 | <0.001 | ω variation for each branch |  |
| **CBS** | Branch site model (Last common ancestral branch of *Balaenopteridae and Eschrichtius robustus*) | | | | | | | |
|  | ma0 | 6313.097 |  |  |  |  | ω0=0.057, ω1= 1, ω2= 1 |  |
|  | ma | 6317.326 | ma0 vs ma | 8.457 | 1 | <0.05 | ω0=0.057, ω1= 1, ω2=999.0 | 299 T 0.994 |
|  | Branch site model (Last common ancestral branch of *Balaenopteridae*) | | | | | | | |
|  | ma0 | 6318.895 |  |  |  |  | ω0=0.057, ω1= 1, ω2= 1 |  |
|  | ma | 6313.702 | ma0 vs ma | 10.387 | 1 | <0.05 | ω0=0.057, ω1= 1, ω2=999.0 | 299 T 0.994 |
| **EIF2AK3** | Branch model (Terminal branch of *Balaena mysticetus)* | | | | | | | |
|  | M0: One ratio | 10027.943 |  |  |  |  | ω **=**0.165 |  |
|  | M1: Free ratio | 9977.553 | M0 vs M1 | 100.778 | 53 | <0.001 | ω variation for each branch |  |
| **FBN1** | Branch model | | | | | | | |
|  | M0: One ratio | 24246.553 |  |  |  |  | ω **=**0.054 |  |
|  | M1: Free ratio | 24127.002 | M0 vs M1 | 239.104 | 51 | <0.001 | ω variation for each branch |  |
| **GALNS** | Branch model | | | | | | | |
|  | M0: One ratio | 5529.435 |  |  |  |  | ω **=**0.076 |  |
|  | M1: Free ratio | 5490.976 | M0 vs M1 | 76.919 | 53 | <0.05 | ω variation for each branch |  |
| **GRB10** | Branch model | | | | | | | |
|  | M0: One ratio | 6935.568 |  |  |  |  | ω **=**0.138 |  |
|  | M1: Free ratio | 6874.677 | M0 vs M1 | 121.783 | 49 | <0.001 | ω variation for each branch |  |
|  | Branch site model (Last common ancestral branch of *Cetacea*) | | | | | | | |
|  | ma0 | 6801.411 |  |  |  |  | ω0 = 0.095 ω1 = 1.0 ω2 = 1.0 |  |
|  | ma | 6797.148 | ma0 vs ma | 8.526 | 1 | <0.05 | ω0=0.099ω1 = 1.0 ω2 =999.0 | 95 G 0.833 |
| **MED12** | Branch model | | | | | | | |
|  | M0: One ratio | 14369.892 |  |  |  |  | ω **=**0.048 |  |
|  | M1: Free ratio | 14325.469 | M0 vs M1 | 88.849 | 53 | <0.001 | ω variation for each branch |  |
| **MEN1** | Branch model | | | | | | | |
|  | M0: One ratio | 5505.936 |  |  |  |  | ω **=**0.075 |  |
|  | M1: Free ratio | 5401.260 | M0 vs M1 | 209.352 | 53 | <0.001 | ω variation for each branch |  |
| **NPR2** | Branch model | | | | | | | |
|  | M0: One ratio | 8243.322 |  |  |  |  | ω **=**0.090 |  |
|  | M1: Free ratio | 8145.060 | M0 vs M1 | 196.525 | 53 | <0.001 | ω variation for each branch |  |
| **OBSL1** | Branch model (Last common ancestral branch of *Tursiops truncatus* and *Delphinus delphis*) | | | | | | | |
|  | M0: One ratio | 17302.035 |  |  |  |  | ω **=**0.106 |  |
|  | M1: Free ratio | 17219.311 | M0 vs M1 | 165.449 | 53 | <0.001 | ω variation for each branch |  |
| **PLAG1** | Branch model | | | | | | | |
|  | M0: One ratio | 5117.959 |  |  |  |  | ω **=**0.046 |  |
|  | M1: Free ratio | 5069.355 | M0 vs M1 | 97.208 | 53 | <0.001 | ω variation for each branch |  |
| **PLOD1** | Branch model | | | | | | | |
|  | M0: One ratio | 6591.421 |  |  |  |  | ω **=**0.065 |  |
|  | M1: Free ratio | 6524.333 | M0 vs M1 | 134.175 | 53 | <0.001 | ω variation for each branch |  |
|  | Branch site model (Terminal branch of *Physeter catodon*) | | | | | | | |
|  | ma0 | 6546.111 |  |  |  |  | ω0 = 0.051 ω1 = 1.0 ω2 = 1.0 |  |
|  | ma | 6541.746 | ma0 vs ma | 8.730 | 1 | <0.05 | ω0=0.051ω1= 1.0 ω2 = 999.0 | 389 T 0.939; 476 R 0.866 |

**Table S4** Radical amino acid sites under positive selection detected by PAML, Datamonkey and TreeSAAP

| **Gene** | **AA**  **position** | **PAML** | **Datamonkey** | | **Clade**  **c** | **AA**  **change** | **TreeSAAP** | |
| --- | --- | --- | --- | --- | --- | --- | --- | --- |
|  |  | **Site**  **Model (M8)^a^** | **FEL (p<0.1^b^)** | **REL**  **(BF>50^b^)** |  |  | **Amino Acid Properties** | **number** |
| **ACAN**  (2022aa) | 9 | 0.996 | 0.042 | 220.159 | t | L→G | *P_α_ B_l_ R_f_ P_c_ F M_v_ V^0^ α_n_  P* | 9 |
|  | 146 |  | 0.059 | 246.616 | e f | L→V |  |  |
|  | 360 | 0.996 | 0.057 | 204.644 | d n w | N→D | *α_c_* | 1 |
|  | 407 |  | 0.093 | 142.754 | e e1 | V→I | *pK’* | 1 |
|  | 703 | 0.995 | 0.069 | 150.907 | w | R→H | *E_sm_* |  |
|  |  |  |  |  | e1 | R→L/L→R | *Ns B_r_ R_f_ h* p *E_sm_ pH_i_ H_nc_ E_t_ α_n_* | 10 |
|  |  |  |  |  | c1 | L→P | *P_α_Ns P_c_ K^0^ F α_c_ α_n_ R_a_ H_p_ P* | 10 |
|  | 975 | 0.999 |  | 130.134 | p o u y e1 | A→T |  |  |
|  | 980 | 0.998 | 0.096 | 136.871 | p u b1 d1 | V→I | *pK’* | 1 |
|  |  |  |  |  | v | V→A |  |  |
|  | 985 | 0.998 | 0.088 | 158.545 | p u b1 d1 | G→R | *C_a_ pH_i_ M_v_* *Mw H_nc_ µ V^0^ E_sm_ E_t_* | 9 |
|  | 1100 | 0.997 |  | 69.418 | v | G→V | *P_β_  B_l_  P_c_  F R_a_  P* |  |
|  | 1734 | 0.934 |  | 55.628 | p u | V→E | *Ns P_β_ B_r_ R_f_ F E_sm_ E_t_α_c_ h* p *H_p_ E_l_ P_r_* | 13 |
|  |  |  |  |  | x | V→M |  |  |
|  | 1944 | 0.912 |  | 135.169 | o v | V→M |  |  |
| **AIP**  (330aa) | 43 | 0.919 | 0.066 | 236.725 | w | S→G |  |  |
|  | 45 | 0.995 |  | 65.856 | h u w | E→D/A | *P* | 1 |
|  | 78 | 0.921 |  | 96.640 | l u | A→C | *Ns B_r_ c C_a_ h pH_i_ H_nc_* p *E_sm_ E_t_* | 10 |
|  | 131 | 0.987 | 0.005 | 293.038 | c | N→S |  |  |
| **CDKN1B** | 162 |  | 0.058 |  | j | S→T |  |  |
| (198aa) |  |  |  |  |  |  |  |  |
| **EIF2AK3**  (964aa) | 154 | 0.966 | 0.093 | 87.548 | k e1 | V→M/I | *P_α_ pK’* | 2 |
|  | 677 | 0.996 |  | 62.123 | j y e1 | I→L/V | *pK’* | 1 |
|  | 897 | 0.929 |  | 138.727 | f | A→C | *Ns B_r_ c C_a_ h pH_i_ H_nc_* p *E_sm_ E_t_* | 10 |
| **FBN1**  (2871aa) | 1946 |  | 0.085 | 176.173 | g | E→V | *Ns B_r_ h F* p *E_t_* | 6 |
|  | 2443 | 0.969 |  | 151.03 | b | I→V | *pK’* | 1 |
|  | 2696 | 0.936 |  | 153.421 | b | S→N |  |  |
|  | 2699 | 0.974 |  | 180.192 | w x | P→L | *P_α_Ns P_c_ K^0^ F α_n_α_c_ R_a_ H_p_ P* | 10 |
|  | 2701 | 0.966 | 0.092 | 193.96 | u p x | A→V | *P_β_* | 1 |
|  | 2737 | 0.985 |  | 163.409 | b | N→D | *α_c_* | 1 |
|  |  |  |  |  | p | D→E | *P* | 1 |
|  | 2741 | 0.999 | 0.024 | 201.773 | c e1 | T→I | *Ns B_r_ pK’ R_a_ H_p_ H_t_* | 6 |
| **MED12**  (2050aa) | 1621 | 0.972 | 0.047 | 207236 | f | Q→R | *pH_i_* | 1 |
|  |  |  |  |  | p | R→Q | *pH_i_* |  |
| **MEN1**  (610aa) | 526 | 0.999 |  | 185.539 | y | A→T | *P_α_* | 1 |
|  | 542 | 0.999 |  | 186.296 | t | A→S | *P_α_ P_c_ P* | 3 |
| **NPR2**  (1047aa) | 325 | 0.999 | 0.080 |  | i | L→V |  |  |
| **NSD1**  (2696aa) | 111 | 0.995 |  | 105.59 | e | I→V | *pK’* | 1 |
|  | 652 | 0.957 |  | 86.368 | j | I→V | *pK’* | 1 |
|  | 964 | 0.943 |  | 92.078 | k | N→S |  |  |
|  | 978 | 0.997 |  | 106.718 | h u | P→S | *α_c_ H_t_* | 2 |
|  | 1493 | 0.963 |  | 96.190 | b | S→N |  |  |
|  | 1518 | 0.955 |  | 102.043 | c | V→I | *pK’* | 1 |
|  | 1853 | 0.968 |  | 115.759 | c | T→A | *P_α_* | 1 |
|  | 2236 | 0.964 |  | 113.588 | c | S→G |  |  |
|  | 2264 | 0.996 |  | 103.976 | v x | T→M | *P_α_* | 1 |
|  | 2335 | 0.995 |  | 96.688 | b | T→R |  |  |
|  | 2411 | 0.950 |  | 95.283 | c | D→E | *P* | 1 |
| **OBSL1**  (1729aa) | 638 | 0.965 |  | 244.23 | w | D→E | *P* | 1 |
|  | 1242 | 0.851 |  | 204.756 | f l | M→V |  |  |
|  | 1395 | 0.869 |  | 235.791 | j | Q→R | *pH_i_* | 1 |
|  | 1428 | 0.910 |  | 82.798 | x | A→T | *P_α_* | 1 |
|  | 1617 | 0.885 |  | 266.03 | x | A→L | *K^0^ P_r_* | 2 |

^a^ Codons identified by PAML as under positive selection along with Bayesian (BEB) analysis PPs for sites with P>85% under M8.

^b^Codons were estimated in Datamonkey.

^c^Amino acid substitution occurred along clades, with detailed information marked in figure 1.

^d^Radical changes in amino acid properties under categories 6-8 were detected in TreeSAAP. Physicochemical amino acid properties available in TreeSAAP are as follows: αc: power to be C-term a-helix; αn: power to be in the N-terminal of an a-helix; Br: buriedness; Ca: helical contact energy; Ei: long-range nonbonded energy; Esm: short- and medium-range nonbonded energy; Et: total nonbonding energy; F: mean r.m.s. fluctuation displacement; h: hydropathy; Hnc: normal consensus hydrophobicity; Hp: surrounding hydrophobicity; Ht: thermodynamic transfer hydrophobicity; Ko: compressibility; μ: refractive index; Mv: molecular volume; Mw: molecular weight; Ns: average number of surrounding residues; Pa: a- helical tendencies; Pβ: b-structure tendencies; Pc: coil tendencies; P: turn tendencies; p: polarity; pHi: isoelectric point; pK’: equilibrium constant of ionization for COOH; Pr: polar requirement; Ra: solvent accessible reduction ratio; RF:chromatographic index; V0: partial-specific volume.

**Table S5** Log likelihood and omega values estimates under different branch models according contracting body length of cetaceans

| **Gene** | **Model** | **-InL** | **LRT** | **Comparisons** | **P value** | **ω value** | | |
| --- | --- | --- | --- | --- | --- | --- | --- | --- |
|  |  |  |  |  |  | **Terrestrial**  **Mammals** | **Large**  **cetaceans** | **Small**  **cetaceans** |
| ***FBN1*** | 1ω | 24246.273 |  |  |  | 0.054 | 0.054 | 0.054 |
|  | 2ω | 24226.259 | 40.028 | 2ω vs 1ω | <0.001 | 0.039 | 0.118 | 0.118 |
|  | 3ω | 24217.475 | 17.569 | 3ω vs 2ω | <0.001 | 0.039 | **0.072** | **0.151** |
| ***GRB10*** | 1ω | 6935.568 |  |  |  | 0.138 | 0.138 | 0.138 |
|  | 2ω | 6907.574 | 55.988 | 2ω vs 1ω | <0.001 | 0.100 | 0.344 | 0.344 |
|  | 3ω | 6904.248 | 6.653 | 2ω vs 3ω | 0.010 | 0.100 | **0.181** | **0.424** |
| ***NPR2*** | 1ω | 8243.322 |  |  |  | 0.090 | 0.090 | 0.090 |
|  | 2ω | 8197.638 | 91.368 | 2ω vs 1ω | <0.001 | 0.043 | 0.316 | 0.316 |
|  | 3ω | 8195.485 | 4.305 | 2ω vs 3ω | 0.038 | 0.043 | **0.172** | **0.375** |
| ***NSD1*** | 1ω | 20944.133 |  |  |  | 0.227 | 0.227 | 0.227 |
|  | 2ω | 20940.386 | 7.495 | 2ω vs 1ω | 0.006 | 0.214 | 0.317 | 0.317 |
|  | 3ω | 20937.958 | 4.856 | 2ω vs 3ω | 0.028 | 0.214 | **0.180** | **0.369** |

**Table S6** Association analysis between gene evolution and phenotypes

| PGLS | | λ | AIC | F-statistic | p | R^2^ | N |
| --- | --- | --- | --- | --- | --- | --- | --- |
| **ACAN** | Body length | 1.00 | 9.138 | 0.025 | 0.876 | 0.002 | 16 |
|  | Body mass | 1.00 | 40.276 | 0.001 | 0.985 | 2.638e-05 | 16 |
| **AIP** | Body length | 0.756 | 8.818 | 2.244 | 0.16 | 0.158 | 14 |
|  | Body mass | 0 | 32.428 | 13.09 | 0.004 | 0.522 | 14 |
| **CBS** | Body length | 0.501 | 4.581 | 7.808 | 0.014 | 0.551 | 16 |
|  | Body mass | 0.597 | 35.555 | 7.414 | 0.017 | 0.561 | 16 |
| **EIF2AK3** | Body length | 1.000 | 8.941 | 0.199 | 0.663 | 0.014 | 16 |
|  | Body mass | 1.000 | 39.720 | 0.496 | 0.493 | 0.034 | 16 |
| **FBN1** | Body length | 1.000 | 9.368 | 0.324 | 0.579 | 0.024 | 16 |
|  | Body mass | 1.000 | 38.189 | 0.939 | 0.350 | 0.067 | 16 |
| **GALNS** | Body length | 1.000 | 8.979 | 0.165 | 0.690 | 0.012 | 16 |
|  | Body mass | 1.000 | 40.070 | 0.182 | 0.676 | 0.013 | 16 |
| **GPC3** | Body length | 1.000 | 8.771 | 0.351 | 0.563 | 0.024 | 16 |
|  | Body mass | 1.000 | 40.119 | 0.010 | 0.715 | 0.010 | 16 |
| **GPR101** | Body length | 1.000 | 9.108 | 0.052 | 0.824 | 0.004 | 16 |
|  | Body mass | 1.000 | 40.271 | 0.005 | 0.946 | 0.001 | 16 |
| **GRB10** | Body length | 1.000 | 6.611 | 3.209 | 0.098 | 0.211 | 14 |
|  | Body mass | 1.000 | 34.647 | 2.991 | 0.109 | 0.200 | 14 |
| **MED12** | Body length | 1.000 | 8.834 | 0.294 | 0.596 | 0.021 | 16 |
|  | Body mass | 1.000 | 39.712 | 0.503 | 0.490 | 0.035 | 16 |
| **MEN1** | Body length | 1.000 | 9.167 | 1.859e-05 | 0.997 | 1.328e-06 | 16 |
|  | Body mass | 1.000 | 40.231 | 0.040 | 0.845 | 0.003 | 16 |
| **NPR2** | Body length | 1.000 | 9.162 | 0.004 | 0.948 | 0.001 | 16 |
|  | Body mass | 1.000 | 40.276 | 0.001 | 0.987 | 1.971e-05 | 16 |
| **NSD1** | Body length | 1.000 | 7.579 | 1.599 | 0.227 | 0.103 | 16 |
|  | Body mass | 1.000 | 38.057 | 3.013 | 0.105 | 0.177 | 16 |
| **OBSL1** | Body length | 1.000 | 6.542 | 2.496 | 0.137 | 0.151 | 16 |
|  | Body mass | 1.000 | 38.027 | 2.113 | 0.168 | 0.131 | 16 |
| **PLAG1** | Body length | 1.000 | 8.499 | 0.597 | 0.453 | 0.041 | 16 |
|  | Body mass | 1.000 | 40.172 | 0.092 | 0.766 | 0.007 | 16 |
| **PLOD1** | Body length | 1.000 | 5.051 | 4.107 | 0.062 | 0.227 | 16 |
|  | Body mass | 1.000 | 37.980 | 2.161 | 0.164 | 0.134 | 16 |
