# Supplementary figures and images for: Insights into body size variation in cetaceans from the evolution of body-size-related genes
Source: BMC Evol Biol. 2019 Jul 27;19:157. doi: 10.1186/s12862-019-1461-9 (PMC6660953; doi:10.1186/s12862-019-1461-9)

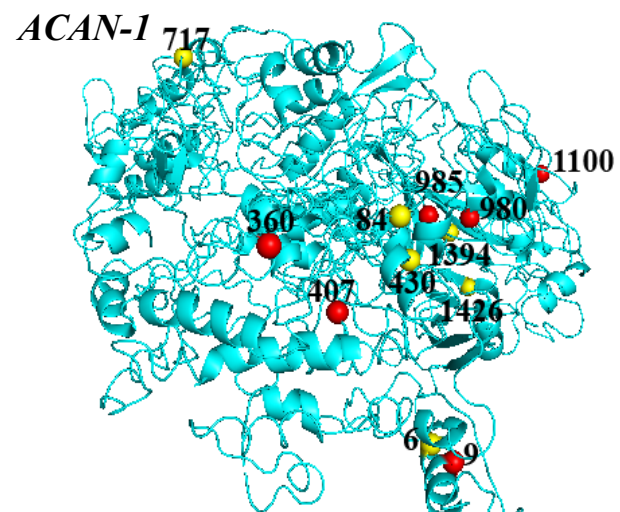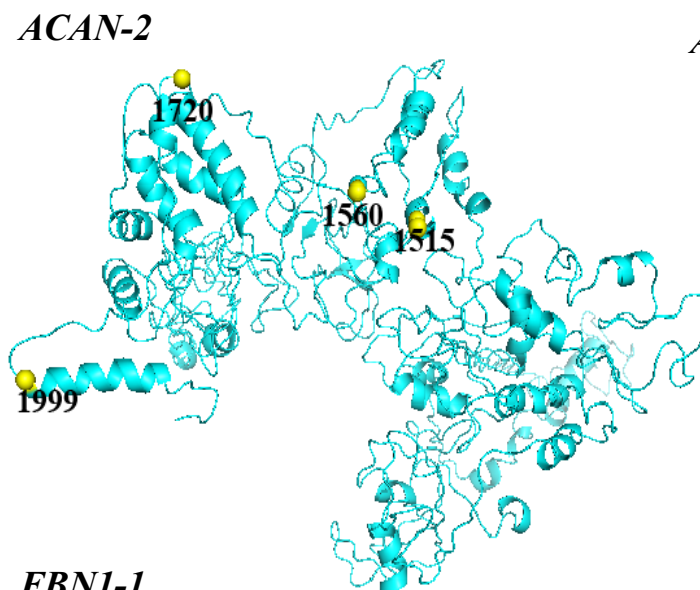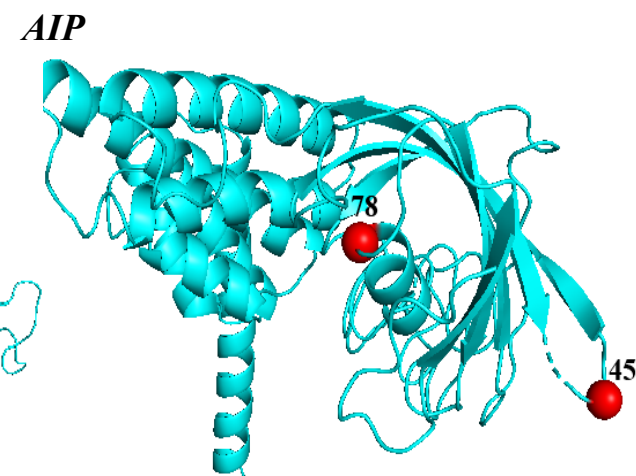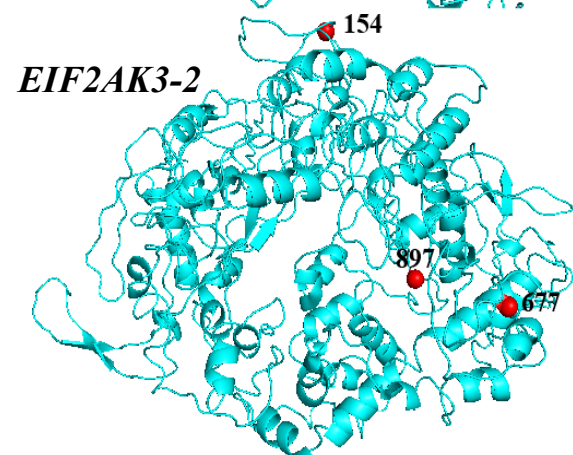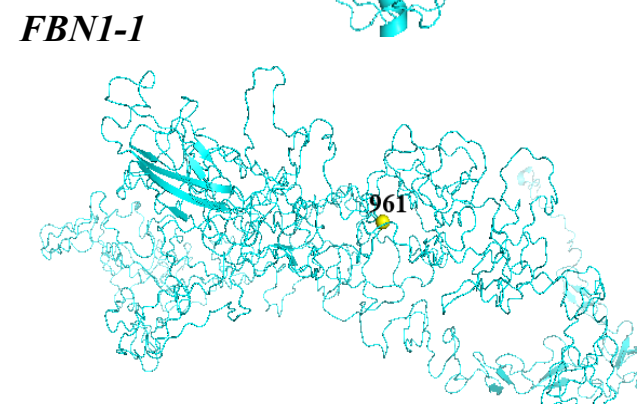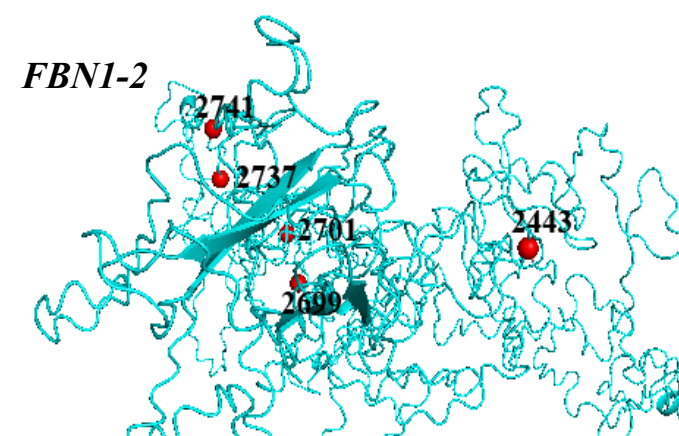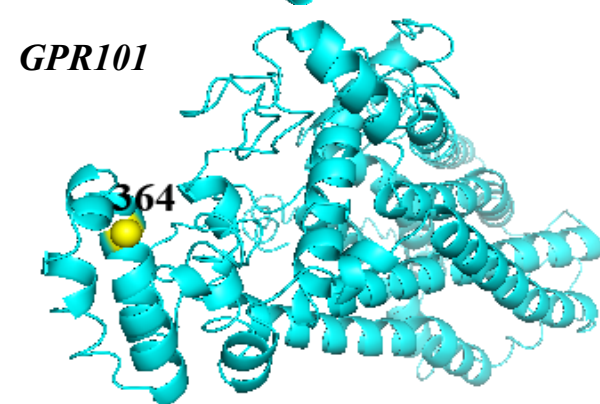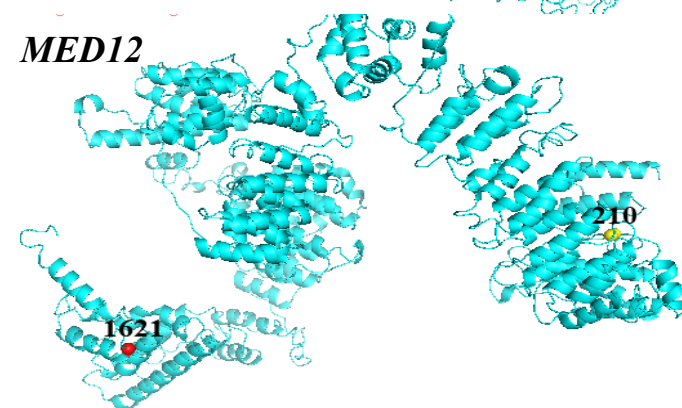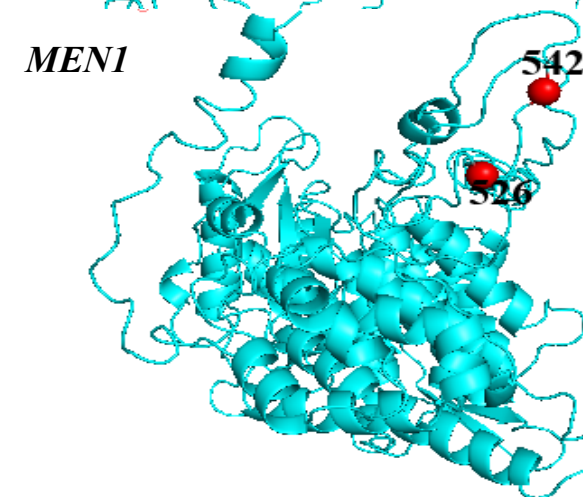

*NPR2*

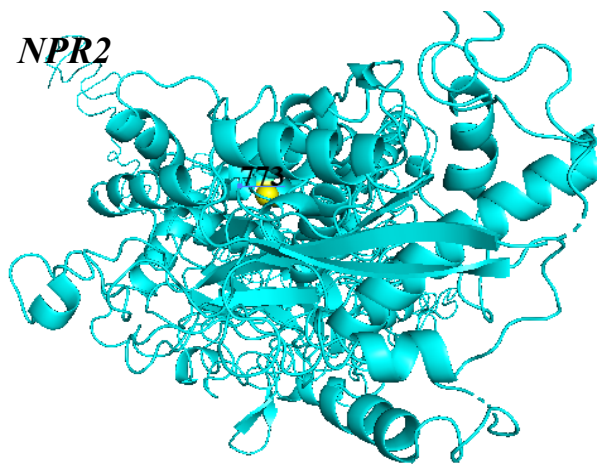

*NSD1-1*

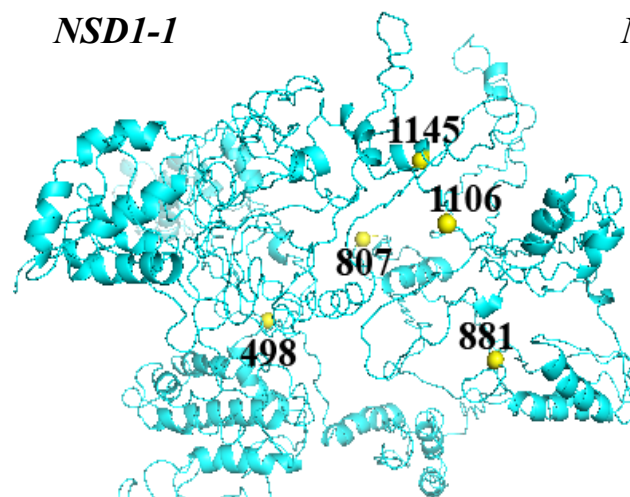

*NSD1-2*

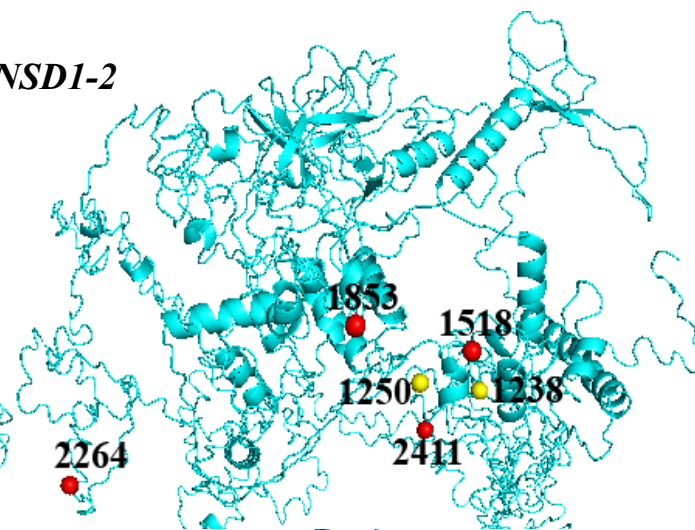

*OBSL1*

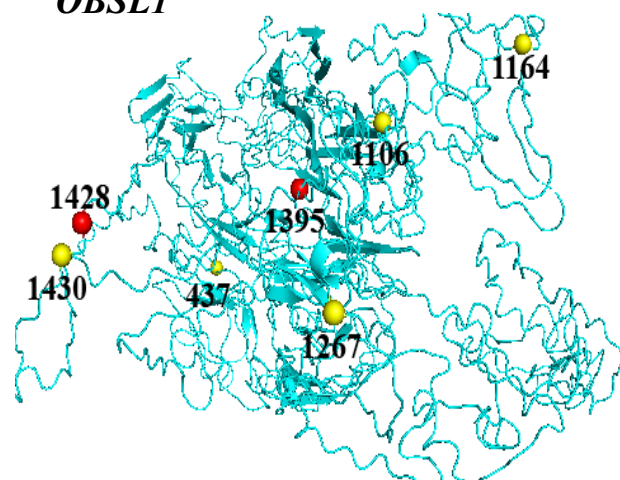

*PLOD1*

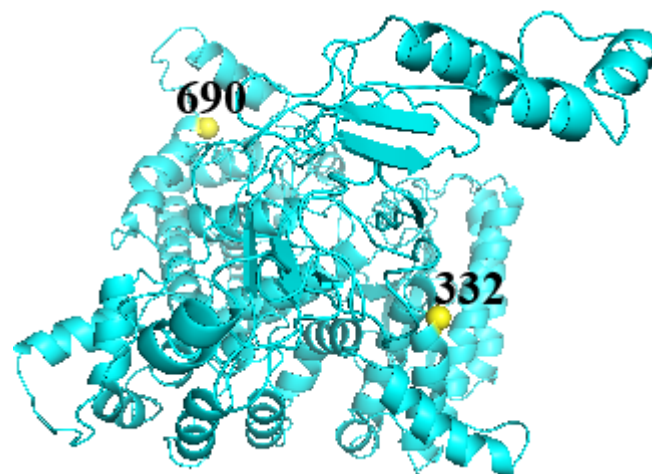

*PLAG1*

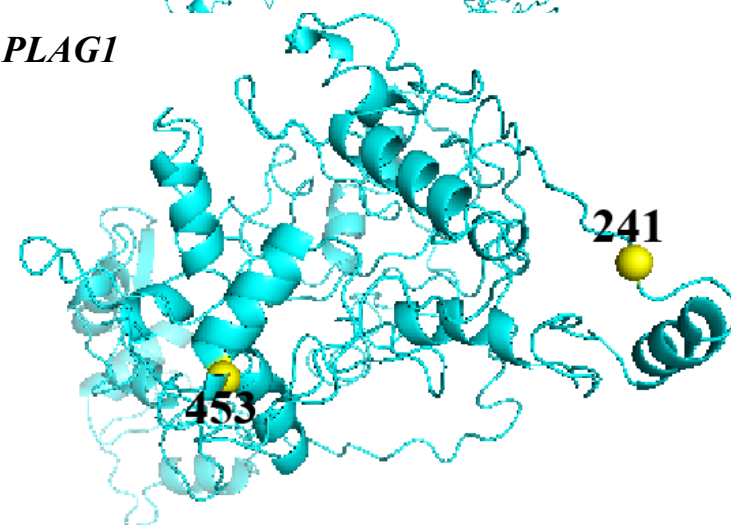

*PIT-1*

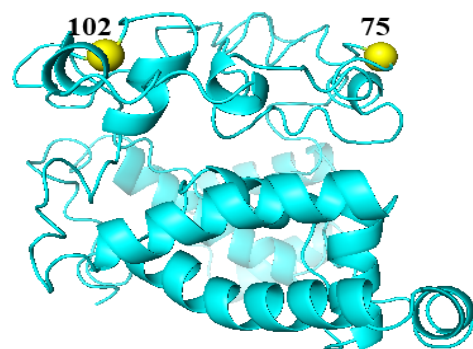

Supplement: Supplementary file 2 — Figure S1. Radical amino acid changes in selected sites and cetacean-special sites mapped on the three-dimensional structure of body-size-related genes. Sites marked with red balls stand for robust sites under selection and yellow balls stand for cetacean-special sites. The figures were created using PyMOL (http://www.pymol.org). (PDF 2961 kb) [file 12862_2019_1461_MOESM2_ESM.pdf]
