# Supplementary material for: Insights into body size variation in cetaceans from the evolution of body-size-related genes
Source: BMC Evol Biol. 2019 Jul 27;19:157. doi: 10.1186/s12862-019-1461-9 (PMC6660953; doi:10.1186/s12862-019-1461-9)
Supplement: Supplementary file 5 — Table S8. Previously reported body size information for cetaceans. (DOCX 17 kb) [file 12862_2019_1461_MOESM5_ESM.docx]

Table S8 Previously reported body size information for cetaceans

| **Maximum body size** | | | | |
| --- | --- | --- | --- | --- |
| **Cetacean species** | **Family** | **m** | **kg** | **References** |
| *Balaena mysticetus* | Balaenidae | 19.8 | 100,000 | Bannister 2002; Rugh & Shelden 2002 |
| *Balaenoptera acutorostrata* | Balaenopteridae | 10.7 | 13,500 | Ohsumi et al. 1970 |
| *Balaenoptera bonaerensis* | Balaenopteridae | 10.7 | 13,500 | Bannister 2002; Reidenberg & Laitman 2002 |
| *Balaenoptera omurai* | Balaenopteridae | 12 |  | Wada et al. 2003 |
| *Eschrichtius robustus* | Eschrichtiidae | 15.2 | 35,000 | Jones & Swartz 2002 |
| *Lipotes vexillifer* | Lipotidae | 2.53 | 160 | Kaiya 2002; Reidenberg & Laitman 2002 |
| *Delphinapterus leucas* | Monodontidae | 5.5 | 150 | O’Corry-Crowe 2002; Reidenberg &Laitman 2002 |
| *Delphinus_delphis* | Phocoenidae | 1.9 | 100 | Reidenberg & Laitman 2002; Amano 2002 |
| *Physeter catodon* | Physeteridae | 20.5 | 57,000 | Reidenberg & Laitman 2002 |
| *Kogia simus* | Physeteridae | 2.7 | 210 | Reidenberg & Laitman 2002 |
| *Orcinus orca* | Delphinidae | 9.75 | 10,500 | Reidenberg & Laitman 2002 |
| *Tursiops truncates* | Delphinidae | 4 | 650 | Reidenberg & Laitman 2002 |
| *Delphinus delphis* | Delphinidae | 2.35 | 235 | Perrin 2002a |
| *Stenella attenuata* | Delphinidae | 2.57 | 119 | Perrin 2002b; Reidenberg & Laitman 2002 |
| *Stenella coeruleoalba* | Delphinidae | 2.4 | 156 | Reidenberg & Laitman 2002 |
| *Grampus griseus* | Delphinidae | 3.35 | 340 | Shoham-Frider et al. 2001 |
